# Character of Cellulase Activity in the Guts of Flagellate-Free Termites with Different Feeding Habits

**DOI:** 10.1673/031.013.3701

**Published:** 2013-04-21

**Authors:** Zhi-Qiang Li, Bing-Rong Liu, Wen-Hui Zeng, Wei-Liang Xiao, Qiu-Jian Li, Jun-Hong Zhong

**Affiliations:** Guangdong entomological institute, Guangzhou 510260, China

**Keywords:** β-glucosidase, endo-β-1, 4-glucanase, fungus-growing termites, humus-feeding termites, Termitidae, wood-feeding termites

## Abstract

Cellulose digestion in termites (Isoptera) is highly important for ecological reasons and applications in biofuel conversion. The speciose Termitidae family has lost flagellates in the hindgut and developed diverse feeding habits. To address the response of cellulase activity to the differentiation of feeding habits, a comparative study of the activity and distribution of composite cellulases, endo-β-1, 4-glucanase, and β-glucosidase was performed in seven common flagellate-free termites with three feeding habits: the humus-feeding termites *Sinocapritermes mushae* (Oshima et Maki), *Malaysiocapritermes zhangfengensis* Zhu, Yang et Huang and *Pericapritermes jiangtsekiangensis* (Kemner); the fungus-growing termites *Macrotermes barneyi* Light and *Odontotermes formosanus* (Shiraki); and the wood-feeding termites *Nasutitermes parvonasutus* (Shiraki) and *Havilanditermes orthonasus* (Tsai et Chen). The results showed that in diverse feeding groups, the wood-feeding group had the highest total composite cellulase and endo-β-1, 4-glucanase activities, while the fungus-growing group had the highest β-glucosidase activity. In terms of the distribution of cellulase activity in the alimentary canals, the cellulase activities in wood-feeding termites were concentrated in the midgut, but there was no significant difference between all gut segments in humus-feeding termites. As for the fungus-growing termites, the main site of composite cellulase activity was in the midgut. The endo-β-1, 4-glucanase activity was restricted to the midgut, but the primary site of β-glucosidase activity was in the foregut and the midgut (*Mac. barneyi*). The functions of the gut segments apparently differentiated between feeding groups. The results suggest that the differentiation of feeding habits in flagellate-free termites was characterized by the distribution of cellulases in the gut rather than by variations in cellulase activity.

## Introduction

Termites (Isoptera) are the most efficient decomposers of cellulose in xylophagous insects and are very important for nutrient cycling in natural ecosystems (Noble et al. 2009; Watanabe and Tokuda 2010). In addition, termites are considered excellent model systems for studying the production of renewable bioenergy (Warnecke et al. 2007; Li et al. 2009). To elucidate the cellulose-digesting mechanisms of termites and provide a reference for the development of cellulosic ethanol, the investigation of cellulases in termites has recently become an important research field (Rubin 2008; Willis et al. 2010).

Currently, there are over 2,600 described termite species around the world, in which Termitidae makes up the bulk of extant termite species (Kambhampati and Eggleton 2000). In Termitidae, the hindgut flagellates have been lost, and the flagellate-free termites have differentiated into diverse feeding-groups based on feeding preferences in response to their ecology (Donovan et al. 2001). Th patterns and characteristics of cellulases in several flagellate-free termites have been studied (Watanabe and Tokuda 2010; Willis et al. 2010; Zeng et al. 2012), but there has never been a comprehensive comparison of the patterns and characteristics of cellulases between the different feeding groups. To provide further information about how cellulase activity responds to the differentiation of feeding habits, the activities and distribution of endo-β1, 4-glucanase (EG; EC 3.2.1.4), β-glucosidase (BG; EC 3.2.1.21), and the composite cellulase (FPase) were compared between seven common flagellate-free termites of China with three different feeding habits, in which *Malaysiocapritermes zhangfengensis* Zhu, Yang et Huang, *Pericapritermes jiangtsekiangensis* (Kemner), *Nasutitermes parvonasutus* (Shiraki), and *Havilanditermes orthonasus* (Tsai et Chen) were first reported.

## Methods and Materials

### Termites

From Termitidae, the humus-feeding termites *Sinocapritermes mushae* (Oshima et Maki) (Termitinae), *Mal. zhangfengensis*, and *P. jiangtsekiangensis* were collected on Luofu Mountain. The fungus-growing termites *Macrotermes barneyi* Light (Macrotermitinae) and *Odontotermes formosanus* (Shiraki) were collected on Luofu Mountain and Maofeng Mountain. The wood-feeding termites *N. parvonasutus* (Nasutitermitinae) and *H. orthonasus* were collected on Dinghu Mountain. The termites are common species in the Guangdong Province of China. Healthy adult workers were selected and placed directly in liquid nitrogen before enzyme extraction. The feeding habits were divided based on the report of Jones and Eggleton (2012). The species were taxonomically identified using Huang et al. (2000).

### Preparation of crude enzyme

Crude enzyme was prepared using the same procedure as described by Zeng et al. (2012). Depending on the number of colonies collected, three (*S. mushae, Mal. Zhangfengensis* and *H. orthonasus*), four (*P. jiangtsekiangensis, Mac. Barneyi* and *N. parvonasutus*), or eight (*O. formosanus*) sets of guts were dissected from worker termites. The guts were divided into foregut (including salivary glands), midgut, and hindgut. The guts were homogenized in 500 µl of 0.1 M sodium acetate buffer (pH 5.6) on ice. After centrifugation at 12,000 rpm for 15 min at 4° C, the supernatants were brought to a volume of 500 µl by adding 0.1 M sodium acetate buffer and used as the enzyme extract. The same volume of 0.1 M sodium acetate buffer was used as the control.

**Table 1.**
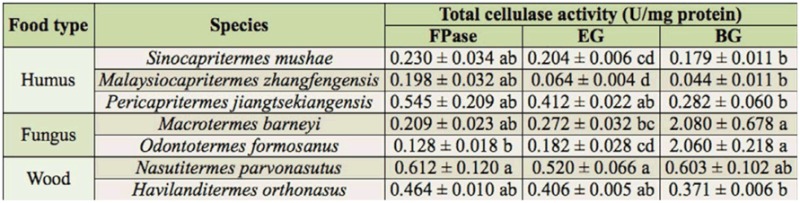
Total cellulase activity in the whole gut of the termite workers. Means ± SE with different lower case letters represent significant differences in the same column using Duncan's multiple range test at the 0.05 probability level. FPase, the composite cellulase; EG, endo-β-1,4-glucanase; BG, β-glucosidase.

### Assay of cellulase activity

The composite cellulase was determined using qualitative filter paper (for chemical analysis, 3.5 mg per piece) as the substrates. The activities of EG and BG were determined using 120 µl of 1% sodium carboxymethyl cellulose and 120 µl of 1% salicin as the substrates, respectively. The determination conditions of cellulase activity previously described were referenced (Mo et al. 2004; Tokuda et al. 2004; Zeng et al. 2012). The substrates were placed into microtubes with 120 µl sodium acetate buffer (pH 5.6) after high temperature sterilization, and the crude enzyme (12 µl) was incubated with filter paper at 37° C for 60 min. Then, 120 µl of dinitrosalicylic acid solution was added, and the mixture was boiled for 5 min and rapidly cooled to room temperature. Glucose production was detected colorimetrically with a Victor 3 Multi-label Microplate Reader (Perkin Elmer, www.perkinelmer.com) at 540 nm, using glucose as a standard. The protein content of the sample was determined spectrophotometrically at 660 nm according to the Coomassie Brilliant Blue G-250 method (Lott et al. 1983), using bovine serum as a standard. One unit of enzyme activity was defined as the amount of enzyme capable of releasing one µmol of reducing sugar per minute. Specific activity was expressed as units per mg protein.

### Data analysis

The data were analyzed by one-way analysis of variance with Duncan's multiple range test using SPSS for Windows (version 13.0, SPSS, Inc., www-01.ibm.com/software/analytics/spss).

## Results

### Total cellulase activities of the guts in termites

**Figure 1.**
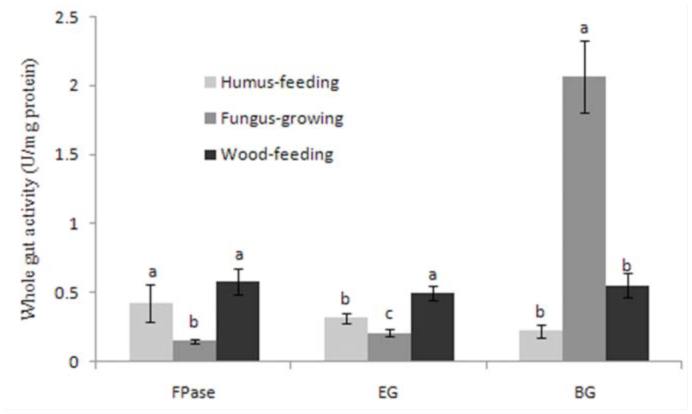
Comparison of the mean total activities of the composite cellulase (FPase), endo-β-1, 4-glucanase (EG), and β-glucosidase (BG) in the different termite feeding groups. Histograms with different letters above them are significantly different (ANOVA and Duncan's multiple test, *p* < 0.05). High quality figures are available online.

The data (Table 1) for the total cellulase activities of whole guts in termite workers showed that the FPase and EG activity of *N. parvonasutus* was the highest of all termites tested, and *Mac. Barneyi* had the highest BG activity. As a whole, the mean FPase activity of the wood-feeding and humus-feeding groups were significantly higher than that of the fungus-growing group, and the wood-feeding group had the highest mean EG activity (Figure 1). However, the fungus-growing group had the highest BG activity and was significantly different from the other feeding groups (Figure 1). Moreover, the significant interspecific differences in the activities of FPase, EG, and BG were neither found between wood-feeding termites nor between fungus-growing termites, but the EG activities were significantly different between *P. jiangtsekiangensis* and the other humus-feeding termites.

**Table 2.**
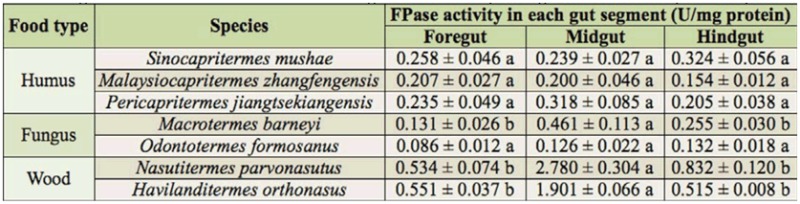
The composite cellulases (FPase) activity of the gut segments in the termite workers. Mean ± SE with different lower case letters means significant difference in the same line using Duncan's multiple range test at the 0.05 probability level.

**Table 3.**
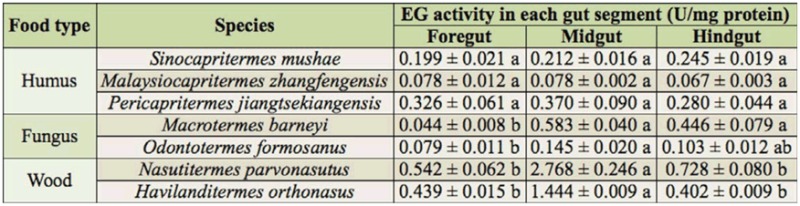
The endo-β-1, 4-glucanase (EG) activity of the gut segments in the termite workers. Mean ± SE with different lower case letters means significant difference in the same line using Duncan's multiple range test at the 0.05 probability level.

**Table 4.**
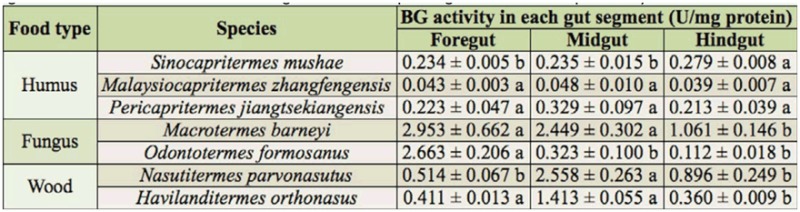
The β-glucosidase (BG) activity of the gut segments in the termite workers. Mean ± SE with different lower case letters means significant difference in the same line using Duncan's multiple range test at the 0.05 probability level.

### Distributions of cellulase activities in termites

**Figure 2.**
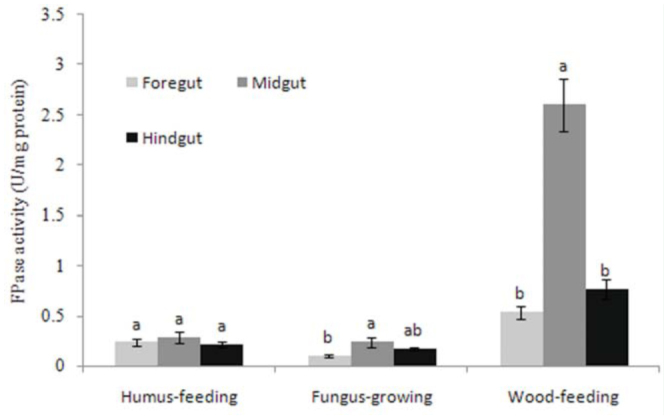
Distribution of mean composite cellulase (FPase) activities in the guts of different termite feeding groups. Histograms with different letters above them are significantly different (ANOVA and Duncan's multiple test, *p* < 0.05). High quality figures are available online.

**Figure 3.**
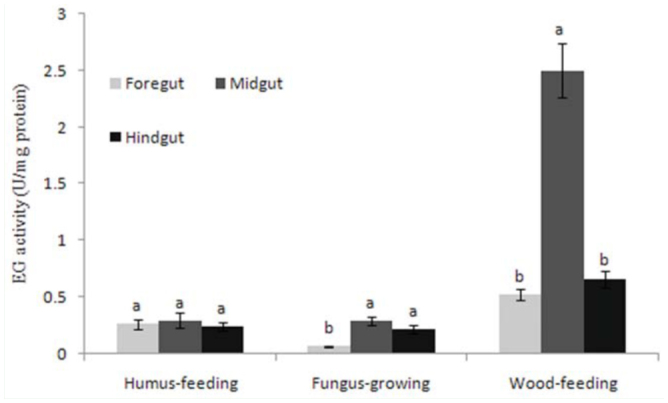
Distribution of mean endo-β-1, 4-glucanase (EG) activities in the guts of different termite feeding groups. Histograms with different letters above them are significantly different (ANOVA and Duncan's multiple test, *p* < 0.05). High quality figures are available online.

Regarding the distribution of FPase and EG activity in the guts of the termites, humus-feeding termites did not show significant differences between the three gut segments, whereas wood-feeding and fungus-growing termites had higher FPase and EG activities in the midgut (Figure 2, 3). However, there were intraspecific differences in the distribution of FPase and EG activities between *Mac. barneyi* and *O. formosanus*. FPase activities among three gut segments of *O. formosanus* showed no significant differences (Table 2, 3).

**Figure 4.**
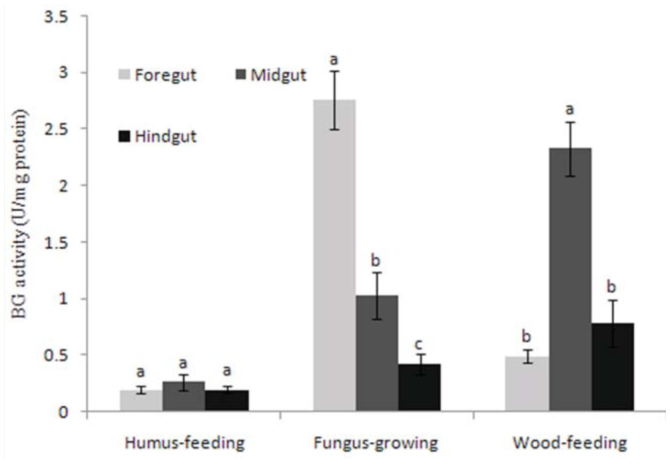
Distribution of mean β-glucosidase (BG) activities in the guts of different termite feeding groups. Histograms with different letters above them are significantly different (ANOVA and Duncan's multiple test, *p* < 0.05). High quality figures are available online.

For distribution of the BG activity, the humusfeeding group as a whole showed no significant difference between segments (Figure 4), but *S. mushae* had relatively high levels of BG activity in the hindgut (Table 4). The midgut was the primary segment in which BG activity was found in the both fungus-growing and wood-feeding termites (Figure 4), and *Mac. barneyi* and *H. orthonasus* were found to have relatively high levels of BG activity both in the foregut and midgut (Table 4).

## Discussion

Special attention has been recently given to the activity and expression of termite cellulases (Watanabe and Tokuda 2010). There are 476 species of termites in China (Huang et al. 2000), and *S. mushae, Mal. zhangfengensis, P. jiangtsekiangensis, Mac. barneyi, O. formosanus, N. parvonasutus*, and *H. orthonasus* are common higher termites in southern China. Wood-feeding termites (*N. takasagoensis, N. walker*, and *N. exitiosus*), fungus-growing termites (*O. formosanus, O. hainanensis*, and *Mac. barneyi*), and the humus-feeding termite (*S. mushae*) have been previously assessed to determine cellulase activities (Lu et al. 2010; Willis et al. 2010; Zeng et al. 2012). However, multinomial factors, such as temperature, substrate, assay method, cellulose-digesting division of worker, etc., are very important when measuring the cellulase activity (Tokuda et al. 2005; Fujita et al. 2008; Willis et al. 2010), so the data are often difficult to compare among studies.

Filter paper, microcrystalline cellulose, and cotton have been used as substrates to determine the existence of complete cellulases (Willis et al. 2010), which have then been used to reflect the composite digestibility of naturally occurring cellulose. The results of the present study indicated that the FPase activities of wood-feeding and humus-feeding termites were markedly higher than that of fungus-growing termites, especially in *N. parvonasutus* and *P. jiangtsekiangensis*. Furthermore, they showed that the midgut was the primary site of FPase activity in woodfeeding termites, which is consistent with the report of Tokuda et al. (2005) for crystalline cellulose hydrolysis. The distribution of total enzymatic activity in the fungus-growing termite *O. formosanus* was no different among the three gut segments, similar to the reports of Tokuda et al. (2005) and Zeng et al. (2012), but the fungus-growing termite *Mac. barneyi* had a higher composite cellulase activity in the midgut.

During the evolution of the Termitidae family, both EG and BG expression shifted from the salivary glands to the midgut (Lo et al. 2012), but differences with regard to the cellulase activity exist between termite species as well as between termite groups. As for EG activity, the present assay confirmed that EG activity in wood-feeding termites was higher than that of fungus-growers, and it was highly concentrated in the midgut (Tokuda et al. 2004; Lo et al. 2011). However, *O. formosanus* was found to have relatively high EG activity in the midgut and hindgut rather than in the salivary glands (Tokuda et al. 2004) or foregut (Mo et al. 2004). In the humus-feeding termite *S. mushae*, EG activities were not significantly different between gut segments, rather than mainly in the midgut as previously reported (Tokuda et al. 2004). In the same way, the BG activity of the wood-feeding termites was most concentrated in the midgut, which agrees with previous reports of BG activity (Tokuda et al. 2004, 2005; Fujita et al. 2008; Tokuda et al. 2009; Lo et al. 2011). Previous reports have also indicated that fungus-growing termites had extraordinarily high BG activity, and that the vast majority of BG activity in the fungus-growing group was distributed in the foregut, including the salivary glands (Zeng et al. 2012). Significant differences of BG activity were not found among gut segments in the humus-feeding group, but the main BG activity of *S. mushae* was in the hindgut.

Despite the low number of termites with different feeding habits that were assayed and compared in this study, it appears that flagellate-free termites with diverse feeding habits degrade cellulose primarily in the midgut. Cellulase activity in flagellate-free termites with different feeding habits showed more differentiation in the gut distributions than variations in cellulase activity.

## Abbreviations

**BG**,: β-glucosidase
**EG**,: endo-β-1, 4-glucanase;
**FPase**,: composite cellulase for filter paper degradation

## References

[bibr01] Donovan S, Jones D, Sands W, Eggleton P (2000). Morphological phylogenetics of termites (Isoptera).. *Biological Journal of the Linnean Society*.

[bibr02] Fujita A, Miura T, Matsumoto T (2008). Differences in cellulose digestive systems among castes in two termite lineages.. *Physiological Entomology*.

[bibr03] Huang FS, Zhu SM, Ping ZM, He XS, Li GX, Gao DR (2000). *Fauna Sinica: Insecta, volume 17: Isoptera*..

[bibr04] Jones DT, Eggleton P, Bignell DE, Roisin Y, Lo N (2011). Global biogeography of termites: a compilation of sources.. *Biology of Termites: a modern synthesis*..

[bibr05] Kambharnpati S, Eggleton P, Abe T, Bignell DE, Higashi M (2000). Taxonomy and phylogeny of termites.. *Termites: evolution, sociality, symbioses, ecology*..

[bibr06] Li X, Yang H, Roy B, Wang D, Yue W, Jiang L, Park EY, Miao Y (2009). The most stirring technology in future: Cellulase enzyme and biomass utilization.. *African Journal of Biotechnology*.

[bibr07] Lo N, Tokuda G, Watanabe H, Bignell DE, Roisin Y, Lo N (2011). Evolution and function of endogenous termite
cellulases.. *Biology of Termites: a modern synthesis*..

[bibr08] Lott J, Stephan VA, Pritchard K (1983). Evaluation of the Coomassie Brilliant Blue G-250 method for urinary protein.. *Clinical chemistry*.

[bibr09] Lu J, Deng T, Li J, Mo J (2010). Activities of some lignocelluloses-degrading enzymes in workers of five common termites (Isoptera).. *Sociobiology*.

[bibr10] Mo J, Yang T, Song X, Cheng J (2004). Cellulase activity in five species of important termites in China.. *Applied Entomology and Zoology*.

[bibr11] Noble JC, Miller WJ, Whitford WG, Pfitzner GH (2009). The significance of termites as decomposers in contrasting grassland communities of semi-arid eastern Australia.. *Journal of Arid Environments*.

[bibr12] Rubin EM (2008). Genomics of cellulosic biofuels.. *Nature*.

[bibr13] Tokuda G, Lo N, Watanabe H, Arakawa G, Matsumoto T, Noda H (2004). Major alteration of the expression site of endogenous cellulases in members of an apical termite lineage.. *Molecular Ecology*.

[bibr14] Tokuda G, Lo N, Watanabe H (2005). Marked variations in patterns of cellulase activity against crystalline- vs. carboxymethylcellulose in the digestive systems of diverse, wood-feeding termites.. *Physiological Entomology*.

[bibr15] Tokuda G, Miyagi M, Makiya H, Watanabe H, Arakawa G (2009). Digestive β-
glucosidases from the wood-feeding higher termite, *Nasutitermes takasagoensis*: Intestinal distribution, molecular characterization, and alteration in sites of expression.. *Insect Biochemistry and Molecular Biology*.

[bibr16] Warnecke F, Luginbühl P, Ivanova N, Ghassemian M, Richardson TH, Stege JT, Cayouette M, McHardy AC, Djordjevic G, Aboushadi N (2007). Metagenomic and functional analysis of hindgut microbiota of a wood-feeding higher termite.. *Nature*.

[bibr17] Watanabe H, Tokuda G (2010). Cellulolytic systems in insects.. *Annual Review of Entomology*.

[bibr18] Willis JD, Oppert C, Jurat-Fuentes JL (2010). Methods for discovery and characterization of cellulolytic enzymes from insects.. *Insect Science*.

[bibr19] Zeng WH, Liu RX, Li ZQ, Liu BR, Li QJ, Xiao WL, Chen LQ, Zhong JH (2012). Comparative lignocellulase activity and distribution among selected termite (Isoptera) genera.. *Journal of Entomological Science*.

